# Autoantibodies neutralizing type I interferons underlie a third of cases of Chikungunya virus encephalitis or myelitis

**DOI:** 10.64898/2026.07.23.26358609

**Published:** 2026-07-27

**Authors:** Vu L. Tran, Adrian Gervais, Marie-Mechtilde Champeaux, Maria Paula de Souza Sampaio, Sylvie Abel, Isabelle Calmont, Mateus Santana do Rosario, Laire Schidlowski, Jordan D. Simione, Pedro Augusto Alves, Anne Puel, Paul Bastard, Laurent Abel, Carolina Prando, Rafael Freitas de Oliveira Franca, Isadora Cristina de Siqueira, André Cabié, Aurélie Cobat, Shen-Ying Zhang, Jean-Laurent Casanova

## Abstract

Chikungunya virus (CHIKV) infection is typically not life-threatening but may, in rare cases, affect the central nervous system (CNS). In three cohorts of confirmed CHIKV cases from Martinique (French overseas territory) and Brazil (patients aged 0–89 years, *n* = 245), 20 patients had CNS infection (aged 17–87 years). Autoantibodies neutralizing type I interferons (AAN-I-IFN) were found in 35% of patients (4 male and 3 female, aged 17–87 years) with encephalitis (4/15), encephalomyelitis (1/2), or myelitis (2/3), but were absent in 225 patients without CNS infection. They neutralized high concentrations (10–1,000 ng/mL) of IFN-α2, IFN-α8, and IFN-ω, and the antibodies of one patient also neutralized IFN-β. All samples also neutralized the other 10 IFN-α subtypes (at least 100 pg/mL). This combination of blood autoantibodies occurs in ∼0.02% and 0.6% of healthy individuals under and over 70 years of age, respectively. The presence of AAN-I-IFN before CHIKV infection therefore increased the risk of CNS disease ∼850-fold relative to the general population.

## Full Text Availability

The license terms selected by the author(s) for this preprint version do not permit archiving in PMC. The full text is available from the preprint server.

